# Mixed Cropping Improves Soil Fertility via Enhancing Plant Facilitation in Alpine Artificial Grassland

**DOI:** 10.3390/biology15171502

**Published:** 2026-09-02

**Authors:** Shengjun Ji, Wei Wang, Mohamed S. Sheteiwy, Youcai Xiong, Fuying Niu, Huimin Li, Wenying Wang

**Affiliations:** 1 College of Geographical Sciences, Qinghai Normal University, Xining 810008, China; 2 State Key Laboratory of Herbage Improvement and Grassland Agro-Ecosystems, College of Ecology, Lanzhou University, Lanzhou 730000, China; 3 Department of Integrative Agriculture, College of Agriculture and Veterinary Medicine, United Arab Emirates University, Al-Ain P.O. Box 15551, United Arab Emirates; 4 Department of Agronomy, Faculty of Agriculture, Mansoura University, Mansoura 35516, Egypt; 5 Qinghai Forestry and Grassland Planning Institute, Xining 810008, China

**Keywords:** plant-plant interaction, biodiversity, soil physical structure, soil fertility, mixed cropping

## Abstract

By enhancing plant complementarity and facilitation, mixed cropping may accelerate soil fertility recovery and improve grassland ecosystem resilience. Nevertheless, the impacts of mixed cropping on soil fertility over a single growing season, and the underlying plant–soil interaction mechanisms, remain poorly understood. We hypothesized that mixed grass–legume–moss sowing would increase above- and belowground biomass, aggregate stability (mean weight diameter), and soil organic carbon and total nitrogen relative to monocultures, partly through relief of the nitrogen limitation of the grass by the nitrogen-fixing legume. To test this hypothesis, seven monoculture and mixed-cropping treatments using *Elymus breviaristatus*, *Medicago sativa*, and *Tortula subulata* were established in May 2024 with three replicate plots per treatment (*n* = 3); plant and soil variables were measured at the end of the growing season (late September 2024), and differences among treatments were tested by one-way ANOVA followed by Tukey’s HSD post hoc test, with significance accepted at *p* < 0.05. Our results showed that compared with monocropping, mixed cropping significantly increased above- and belowground biomass, improved soil water content, and reduced bulk density, thereby creating a more favorable soil microenvironment. Compared with monocropping, mixed cropping enhanced microbial biomass C and N by 26% and 10%, respectively, and increased soil organic C by 30%. Fertility indices were positive in all mixed-cropping systems, contrasting with the negative values observed for two of the three monocultures and for the unseeded control. Across treatments, the facilitation index was positively correlated with the fertility index (R^2^ = 0.757), indicating that interspecific facilitation was closely associated with soil fertility gains. These findings suggest that mixed cropping can enhance soil fertility restoration in alpine artificial grassland and that this effect is consistent with interspecific facilitation promoting biomass accumulation, improving soil physicochemical conditions, and supporting soil microbial biomass. These results highlight species diversification as a promising strategy for accelerating soil fertility restoration, although multi-year and multi-site validation is still required.

## Simple Summary

Mixed cropping serves as a promising restoration approach for degraded alpine artificial grasslands. However, the effects of mixed-cropping plant assemblages on soil fertility over a single growing season, and the underlying plant–soil interaction mechanisms, remain poorly elucidated. This field experiment was conducted in Haiyan County, Haibei Prefecture, Qinghai Province, on the Qinghai–Xizang Plateau in 2024, where we established monoculture and mixed cropping treatments using *Elymus breviaristatus*, *Medicago sativa*, and *Tortula subulata*. We quantified above-and belowground biomass, soil physicochemical properties, microbial nutrients, and fertility indices to clarify how interspecific interactions modulate soil conditions. Mixed cropping enhanced plant biomass, optimized soil physical microhabitats, and elevated soil organic carbon and microbial biomass relative to monocultures and the unseeded control. A strong positive correlation between the facilitation index and the fertility index (R^2^ = 0.757) indicated that interspecific facilitation was closely associated with the improvement of soil fertility. This work provides preliminary, single-season evidence that mixed cropping is associated with accelerated soil restoration, offering a candidate strategy for the rehabilitation of degraded alpine grasslands.

## 1. Introduction

Alpine meadow, as an important component of terrestrial ecosystems, plays a pivotal role in global carbon (C) and nitrogen (N) cycling, soil and water conservation, biodiversity maintenance, and livestock production [1,2,3,4]. Globally, grasslands cover nearly 40% of the Earth’s land surface [5] and are recognized as critical C sinks that can mitigate climate change while supporting multiple ecosystem services [6]. On the Qinghai–Xizang Plateau, one of the highest and most fragile regions in the world, alpine meadows are the dominant grassland type and play a critical role in regional climate regulation, water conservation, and pastoral production [7,8,9,10]. Despite this importance, grassland integrity is declining under converging pressures from climate change, overgrazing, and inappropriate land use [11,12]. These stressors drive widespread degradation characterized by reduced vegetation coverage, biodiversity loss, disrupted soil structure, and declining fertility, which in turn weaken the ecosystem service functions and ecological resilience of alpine grasslands [13,14,15,16]. Artificial planting of grass and legume forages currently represents the dominant restoration strategy for severely degraded grasslands across the Qinghai–Xizang Plateau, and restoring degraded grasslands is essential for stabilizing and enhancing ecosystem services while safeguarding co-benefits for biodiversity and livelihoods [17]. However, monoculture sowing, the most widely used restoration practice on the plateau, is often constrained by low community stability, unbalanced nutrient acquisition, and limited belowground niche complementarity, which restrict the long-term effectiveness of vegetation restoration for soil fertility recovery.

Mixed cropping (or intercropping) has emerged as a potentially more effective approach for accelerating ecosystem recovery in degraded grasslands. By combining plant species with complementary niches and facilitative interactions, mixed communities can enhance nutrient acquisition, increase primary productivity, and improve soil physical and biochemical properties [18]. Plant facilitation, a key ecological process that regulates resource allocation within plant communities and drives their overproduction, refers to positive interactions in which one plant improves the performance or the environmental conditions of another plant [19]. It should be noted, however, that although mixed cropping ensures favorable development for at least one companion plant, the interactions among the component species are not necessarily facilitative and may also include competition. Niche complementarity, in contrast, refers to the partitioning of limiting resources among species with different traits; both processes can contribute to the overyielding of mixed communities. For instance, in grass–legume associations, the grass is usually the dominant partner owing to its initial developmental advantage; as the grass rapidly depletes the soil nutrient and water reserves, the legume increasingly fixes atmospheric nitrogen, thereby functioning as a “nitrogen pool” that benefits the companion grass, while the rapid growth of grasses promotes the accumulation of soil C [20,21]. Although plant facilitation is common within plant communities, it remains unclear whether applying its fundamental principles can enhance ecosystem functions in mixed-species communities.

The study species were selected to represent the main functional groups involved in these processes. Combinations of *Elymus breviaristatus* (a perennial grass with strong soil-holding capacity) and *Medicago sativa* (a legume capable of symbiotic N fixation) have been widely used to improve soil fertility and vegetation stability [22,23,24]. Biological soil crusts (BSCs) are soil-surface communities composed of cyanobacteria, algae, lichens, mosses, fungi, and other microorganisms closely associated with soil particles; they can cover the ground surface, effectively reducing soil water evaporation and soil erosion [25]. Introducing BSCs, such as moss, into mixed cropping systems may promote soil nutrient retention by secreting polysaccharides to facilitate soil aggregate formation, or enhance soil water content by covering the soil to reduce soil water evaporation [26]. These advantages could potentially facilitate the productivity of mixed cropping systems and cascade to affect soil fertility [27].

Despite these potential synergies, the interactive mechanisms by which plant mixtures and BSCs jointly influence soil fertility and ecosystem functions remain poorly understood. Most existing studies have focused on either mixed cropping or BSC effects in isolation, while few have explored their combined impacts on plant–soil feedbacks and the short-term restoration of degraded alpine meadow. Unraveling these mechanisms is therefore crucial for developing optimized mixed cropping models tailored to the unique ecological conditions of the Qinghai–Xizang Plateau and for advancing the theory and practice of grassland restoration in cold, arid regions [28]. Therefore, this study focused on alpine restored grasslands artificially established on severely degraded alpine meadow substrates; we established seven treatments: (1) unseeded control (S0); (2) monocropping of *Elymus breviaristatus* (S1); (3) monocropping of *Medicago sativa* (S2); (4) monocropping of *Tortula subulata* (S3); (5) mixed cropping of *Elymus breviaristatus* and *Tortula subulata* (S4); (6) mixed cropping of *Medicago sativa* and *Tortula subulata* (S5); and (7) mixed cropping of *Elymus breviaristatus* and *Medicago sativa* (S6). The specific objectives of this study were to (1) quantify the effects of monoculture and mixed-cropping patterns on plant biomass and on soil C, N, and P pools over a single growing season; (2) determine whether legume–grass–moss mixed cropping improves soil physical properties (aggregate stability and bulk density) and microbial biomass (MBC and MBN) relative to monocultures; and (3) assess the extent to which interspecific facilitation is associated with the restoration of soil fertility. The research results will provide theoretical support for the rapid restoration of alpine meadow and fill the research gap in the synergistic enhancement of legume–grass–moss interactions.

## 2. Materials and Methods

### 2.1. Experimental Location

**Figure 1:**
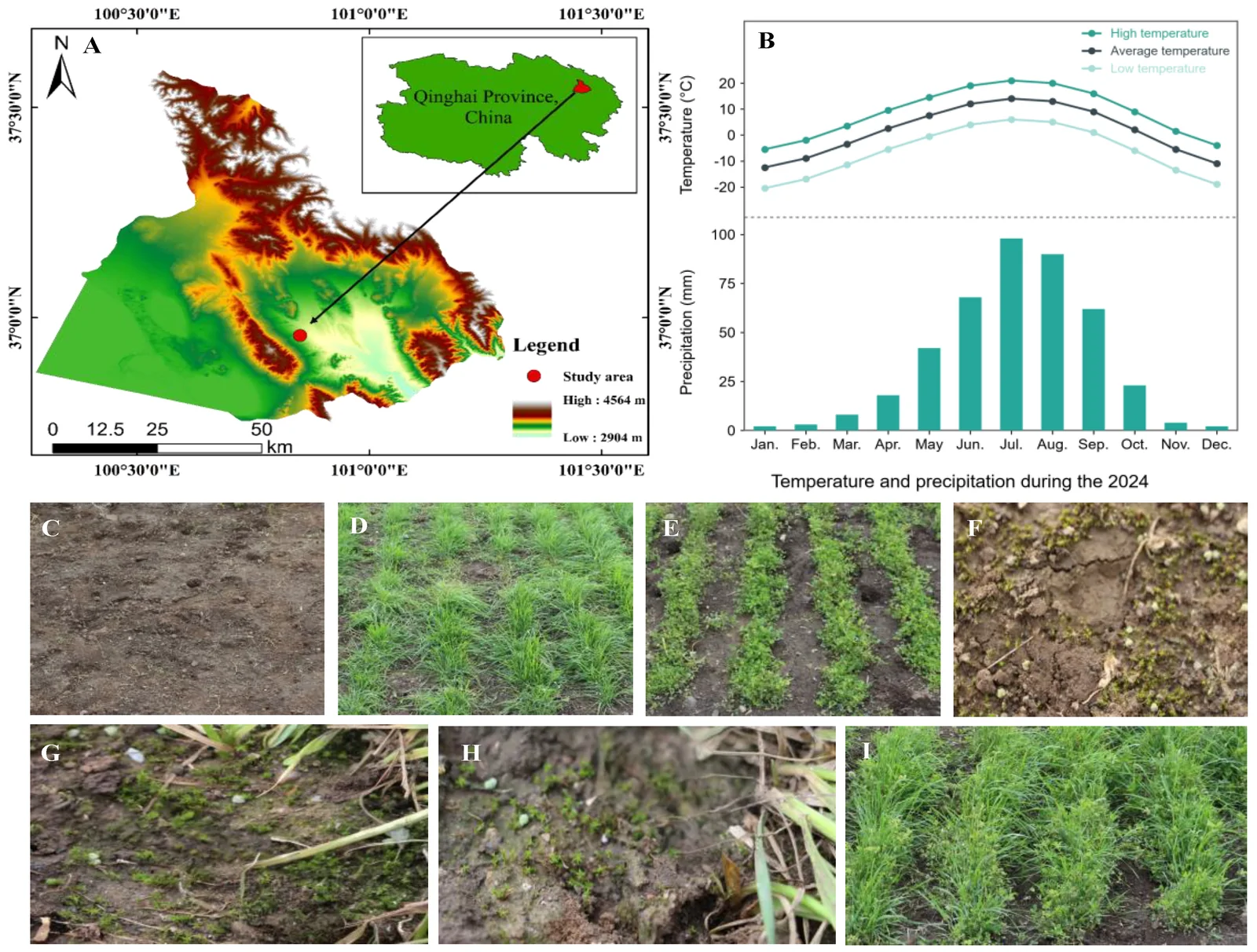
Schematic diagrams of the meteorological data and the planting pattern in each plot. (**A**) Location of the study area and the sampling field. (**B**) Monthly mean temperature and precipitation during 2024; the total annual precipitation in 2024 was 420 mm. (**C**–**I**) Planting patterns of the unseeded control (S0), the *Elymus breviaristatus*, *Medicago sativa*, and *Tortula subulata* monocultures, the *Elymus breviaristatus*–*Tortula subulata*, *Medicago sativa*–*Tortula subulata*, and *Elymus breviaristatus*–*Medicago sativa* mixtures, and the corresponding field photographs. The dashed line denotes the boundary separating the two y-axes (dual-axis plot). Photographs taken by the authors.

The study site was located in Xihai Town, Haiyan County, Haibei Tibetan Autonomous Prefecture, Qinghai Province, China (36°44′ N, 100°23′ E; 3140 m a.s.l.). The region has a plateau continental climate with relatively low precipitation and high evaporation. Climatic data were obtained from the Haiyan County Meteorological Station (Xihai Town), close to the study site. Over the past 10 years (2014–2023), the mean annual temperature ranged from −1.5 to 1.7 °C, the mean annual precipitation was approximately 400 mm (concentrated from June to September), and the mean annual pan evaporation was approximately 1538 mm. In 2024, the total annual precipitation was 420 mm, slightly above the long-term average, and the mean annual temperature was approximately 1.5 °C; the monthly temperature and precipitation during the 2024 growing season are shown in Figure 1B. The zonal climax vegetation is alpine steppe-meadow, a transitional vegetation type between alpine steppe and alpine meadow, dominated by *Kobresia pygmaea*, *Kobresia humilis*, and *Elymus nutans* as determined by a pre-sowing vegetation survey based on relative canopy cover. The soil is classified as a Calcaric Cambisol according to the FAO World Reference Base for Soil Resources [29], corresponding to alpine meadow soil in the Chinese Soil Taxonomy. The experimental site is flat to gently sloping (inclination < 5°), with a uniform microrelief and no pronounced slope aspect. The site had been used as a moderately grazed winter pasture for more than 20 years, and degradation (patchy vegetation cover and declining productivity) had been observed for approximately 10 years before the experiment. The site was selected because it is representative of the degraded alpine meadows widely distributed in the study region and because its uniform topography and consistent management history minimized background heterogeneity. Before the experiment, the topsoil (0–20 cm) had the following properties (mean ± SD, *n* = 3): pH 8.22 ± 0.06, soil organic carbon (SOC) 16.18 ± 0.52 g kg^−1^, total nitrogen (TN) 3.18 ± 0.11 g kg^−1^, available phosphorus (AP) 1.54 ± 0.09 mg kg^−1^, and bulk density (BD) 1.45 ± 0.03 g cm^−3^.

### 2.2. Experimental Design and Sample Plot Management

This study employed a randomized block experimental design with seven treatments: (1) unseeded control (S0; the S0 plots were tilled and prepared in the same way as the sown plots but received neither seeds nor moss propagules, and they remained bare ground throughout the experiment); (2) monocropping of *Elymus breviaristatus* (S1); (3) monocropping of *Medicago sativa* (S2); (4) monocropping of *Tortula subulata* (S3); (5) mixed cropping of *Elymus breviaristatus* and *Tortula subulata* (S4); (6) mixed cropping of *Medicago sativa* and *Tortula subulata* (S5); and (7) mixed cropping of *Elymus breviaristatus* and *Medicago sativa* (S6). Each treatment was replicated three times, resulting in 21 plots in total. The area of each plot was 3 m × 2 m, with a spacing of 1 m between quadrats to prevent border (row) effects. The three blocks were laid out across the site so that each block was as internally homogeneous as possible with respect to topography, soil moisture, and the initial state of degradation; within each block, the seven treatments were randomly assigned to plots, with one plot per treatment per block. Seeding was conducted using a strip sowing method. *Elymus breviaristatus* and *Medicago sativa* were sown in May 2024 at the locally recommended rates of 22.5 g m^−2^ for *Elymus breviaristatus* and 15 g m^−2^ for *Medicago sativa*. The sowing depth was 10 cm, with a row spacing of 20 cm. Two months prior to sowing, *Tortula subulata* approximately 1 cm thick was collected near the experimental site and brought back to the laboratory. After being dried in the shade, it was crushed and sieved through a 2 mm sieve to remove weeds and root fragments. After the canopy of *Elymus breviaristatus* and *Medicago sativa* attained adequate light interception, *Tortula subulata* propagules were uniformly distributed over the topsoil via manual transplantation, applied at 1 kg per plot. During the plant growth period, weeds were removed, and uniform field management was conducted. No fertilization treatment was applied to the plots.

### 2.3. Sampling and Determination

Sampling was conducted in late September 2024, at the end of the single growing season. All above-ground plant parts were cut from a 0.5 m × 0.5 m quadrat in each plot. The aboveground plant material was oven-dried at 105 °C for 30 min to inactivate enzymes and then dried at 80 °C to constant weight before weighing, following the conventional oven-drying protocol for plant biomass determination. Root sampling was restricted to the 0–20 cm soil layer. The collected roots were cleaned with water, dried, and weighed. Soil samples were collected using the root core method. Specifically, three soil cores (5 cm in diameter, 0–20 cm deep) were collected from randomly selected locations and homogenized together after removing surface litter, and then passed through a 2 mm sieve to remove the fine root fragments. Each treatment had three independent plot replicates (*n* = 3). The three soil cores from each plot were pooled into one composite sample, so that the plot (not the individual core) was the experimental unit in all statistical analyses. The collected soil samples were divided into two parts: one was stored at 4 °C as a bulk (rooting-zone) soil sample for measuring microbial biomass C and N (MBC and MBN) and inorganic N (NH_4_^+^-N and NO_3_^−^-N), while the other was naturally air-dried for analyzing soil organic C (SOC), total nitrogen (TN), total P, available P, pH, and aggregate distribution. The contents of SOC and TN were measured using an elemental analyzer (Elementar Analysensysteme GmbH, Langenselbold, Germany). The contents of soil MBC and MBN were determined using the chloroform fumigation method [30]. Specifically, fresh soil samples equivalent to 10 g of oven-dried soil were divided into two equal parts. The first part was fumigated for 24 h and then extracted with 0.5 mol L^−1^ K_2_SO_4_. The second one was non-fumigated as a control, followed by extraction with the same K_2_SO_4_. The MBC and MBN contents were determined as the difference between the C and N contents in fumigated and non-fumigated soil extracts using a vario TOC cube analyzer (Elementar Analysensysteme GmbH, Langenselbold, Germany) with corrections for conversion factors KC and KN of 0.45 and 0.54 [31]. The above unfumigated soil extracts were further used for the determination of soil NH_4_^+^-N and NO_3_^−^-N by using a FIAstar 5000 Analyser (FOSS Tecator AB, Höganäs, Sweden). Soil total P was determined colorimetrically after perchloric acid digestion, while available P (AP) was determined using the Olsen method. Soil pH was determined in a soil: water suspension of 1:2.5. Soil moisture content (SWC %) was determined using the weighing method. The wet sieving method was used to separate water-stable soil aggregates [32]. These water-stable aggregates of different sizes were weighed to measure the mean weight diameter (MWD), which was used as an index of soil aggregate stability. Soil bulk density (BD) was determined by collecting soil at 15 cm depth using a cutting ring. The soil fertility index integrates soil organic carbon, total nitrogen, available phosphorus, and bulk density to comprehensively evaluate soil structural properties and nutritional status across different sowing treatments. The interspecific facilitation index quantifies the intensity of positive interspecific interactions, characterizing niche complementarity and resource partitioning within mixed plant communities.

### 2.4. Data Processing

Differences among the seven treatments were first tested for all parameters by one-way analysis of variance (ANOVA) followed by Tukey’s HSD post hoc test, with statistical significance accepted at *p* < 0.05; *p* < 0.01 and *p* < 0.001 are indicated where applicable in the figures. In addition, the treatments were pooled into monoculture and mixed-cropping groups, and differences between the two groups were examined using independent-samples *t*-tests.

The comprehensive soil fertility index was computed from four measured indicators, namely soil organic carbon (SOC), total nitrogen (TN), available phosphorus (AP), and bulk density (BD). Each indicator was standardized by z-score normalization to eliminate dimensional differences, and the standardized score of bulk density was multiplied by −1, because higher bulk density indicates poorer soil physical conditions. The fertility index was then calculated as the equally weighted sum of the four standardized scores; consequently, the index has no absolute scale; its zero point corresponds to the mean standardized fertility level across all treatments, and negative values indicate fertility below the experimental mean rather than an absence of fertility. In addition, we employed linear fitting to examine the relationship between the fertility index and the facilitation index, and utilized heatmaps and Pearson’s test to analyze the correlation between plant productivity and various soil properties. All statistical analyses were conducted using SPSS 22 (SPSS Inc., Chicago, IL, USA), with a significance level set at *p* < 0.05. The charts were plotted using Origin 2021 software (OriginLab Corporation, Northampton, MA, USA).

## 3. Results

### 3.1. The Impact of Different Treatments on the Plant Aboveground and Belowground Biomass

**Figure 2:**
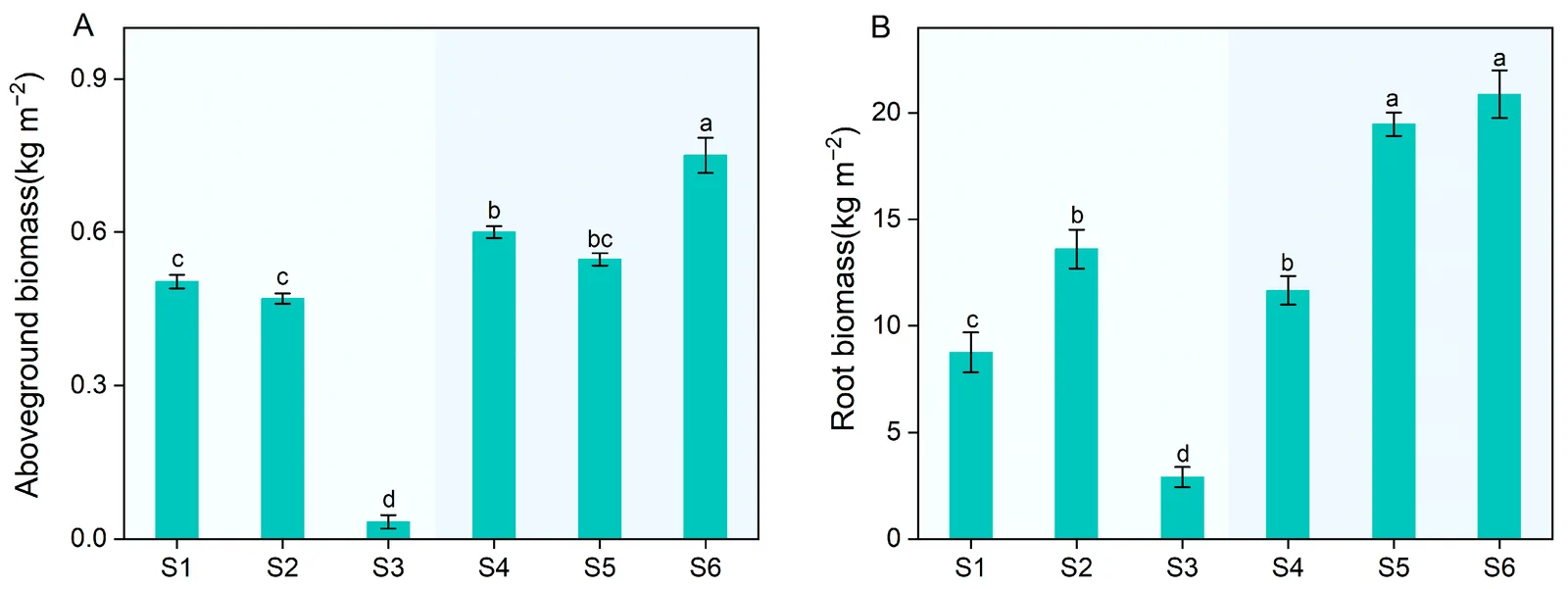
Aboveground biomass (**A**) and root biomass (**B**) under the different treatments in the 2024 growing season. S1, S2, and S3 refer to the *Elymus breviaristatus*, *Medicago sativa*, and *Tortula subulata* monocultures; S4, S5, and S6 refer to the *Elymus breviaristatus*–*Tortula subulata*, *Medicago sativa*–*Tortula subulata*, and *Elymus breviaristatus*–*Medicago sativa* mixtures, respectively. Different lowercase letters indicate significant differences among treatments at *p* < 0.05 (*n* = 3). The shaded background bands visually separate the monoculture treatments (S1–S3) from the mixed-cropping treatments (S4–S6).

The unseeded control (S0) remained bare ground throughout the experiment, and no aboveground or belowground biomass data were collected from this treatment. The aboveground biomass was statistically indistinguishable between *Elymus breviaristatus* (S1, 0.50 kg m^−2^) and *Medicago sativa* (S2, 0.47 kg m^−2^) (*p* > 0.05; Figure 2). *Tortula subulata* monoculture (S3) exhibited the lowest aboveground biomass across all treatments. Among mixed-cropping assemblages, the *Elymus breviaristatus*–*Medicago sativa* mixture (S6) achieved the maximum aboveground biomass (0.75 kg m^−2^), followed by the *Elymus breviaristatus*–*Tortula subulata* mixture (S4, 0.60 kg m^−2^) and the *Medicago sativa*–*Tortula subulata* mixture (S5, 0.55 kg m^−2^).

Root biomass displayed an identical treatment ranking to aboveground biomass. The *Elymus breviaristatus*–*Medicago sativa* mixture (S6) sustained the highest root biomass (20.5 kg m^−2^). Root biomass was comparable between monocultures of *Elymus breviaristatus* (S1) and *Medicago sativa* (S2); both values were significantly lower than those of S6, whereas *Tortula subulata* monoculture (S3) yielded the minimum root biomass. Within the mixed communities, root biomass was numerically higher in S4 than in S5, but this difference was not significant (*p* > 0.05). Among the mixtures, S4 and S6 showed significantly greater aboveground and root biomass than the three monocultures, whereas S5 did not differ significantly from the monocultures S1 and S2 (*p* > 0.05). The consistent ranking of aboveground and root biomass across treatments suggests coordinated shoot and root growth responses to vegetation composition, indicating that appropriate species mixing can improve plant biomass production under alpine restoration conditions.

### 3.2. The Impact of Different Treatments on Soil Fertility

**Figure 3:**
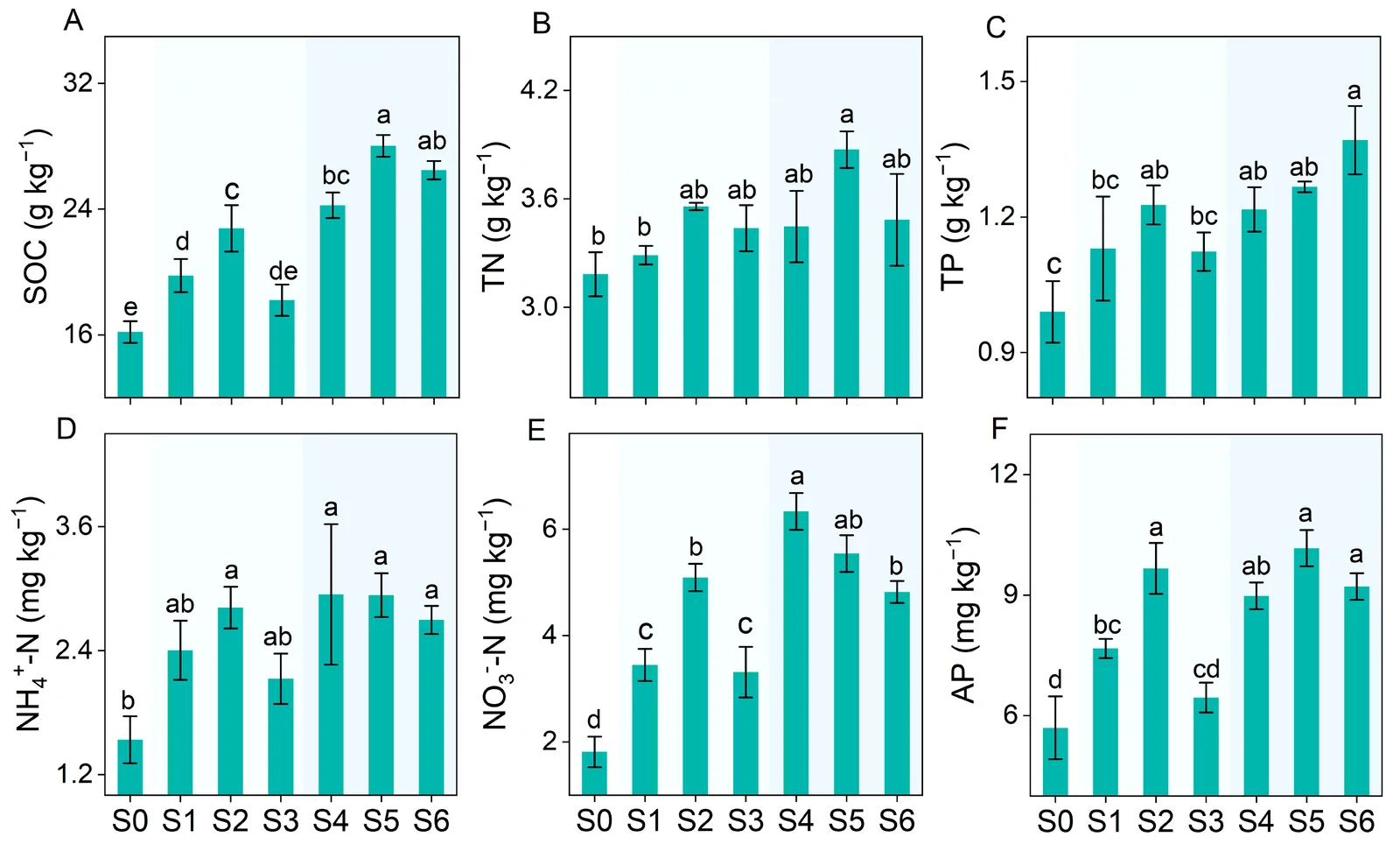
Soil organic carbon (SOC) (**A**), total nitrogen (TN) (**B**), total phosphorus (TP) (**C**), ammonium nitrogen (NH_4_^+^-N) (**D**), nitrate nitrogen (NO_3_^−^-N) (**E**), and available phosphorus (AP) (**F**) under the different treatments in the 2024 growing season. S0 refers to the unseeded control treatment. Different lowercase letters indicate significant differences among treatments at *p* < 0.05 (Tukey’s HSD test, *n* = 3). The shaded background bands visually separate the unseeded control and monoculture treatments from the mixed-cropping treatments.

Overall, mixed-cropping assemblages accumulated significantly higher soil organic carbon (SOC) than the monoculture treatments (*p* < 0.05; Figure 3), whereas the responses of total nitrogen (TN) and total phosphorus (TP) were less consistent and significant only for individual treatments. All mixed-cropping treatments yielded elevated SOC levels (24.24 g kg^−1^, 28.02 g kg^−1^, and 26.47 g kg^−1^), which were consistently greater than those of monocultures (19.77 g kg^−1^, 22.77 g kg^−1^, 18.21 g kg^−1^) and the unseeded control (S0, 16.18 g kg^−1^). Specifically, S5 and S6 exhibited significantly higher SOC relative to both monoculture and control treatments. In contrast, treatment effects on TN were relatively weak, with only S5 (3.87 g kg^−1^) showing a significant increase relative to the unseeded control S0 (3.38 g kg^−1^), whereas S5 did not differ significantly from S2 (3.59 g kg^−1^) (*p* > 0.05). TP exhibited a similar marginal response, where significant differences were only detected between S6 (1.37 g kg^−1^), S2 (1.13 g kg^−1^), and S4 (1.21 g kg^−1^), while no statistical divergence was observed for the remaining treatments.

In terms of soil available nutrients, ammonium nitrogen (NH_4_^+^−N) differed significantly among treatments (*p* < 0.05; Figure 3D), and the detailed pairwise groupings are indicated by the letter codes in Figure 3D. However, nitrate nitrogen concentrations were consistently elevated under mixed-cropping systems (4.82–6.33 mg kg^−1^), with all mixed-cropping treatments showing significantly higher nitrate nitrogen than S0 (1.81 mg kg^−1^), S1 (3.45 mg kg^−1^), and S3 (3.31 mg kg^−1^). Similarly, mixed cropping generally increased soil available phosphorus, although the difference was not statistically significant relative to S2 (9.67 mg kg^−1^). Across all nutrient indices, the unseeded control exhibited the lowest soil nutrient status. Collectively, these results indicate that the benefits of mixed cropping for soil nutrient status are clearest for SOC and nitrate nitrogen, whereas the effects on TN, TP, and available phosphorus are indicator-specific and less consistent. The differential responses of total and available soil nutrients indicate that vegetation composition regulates soil nutrient status in a component-specific manner.

### 3.3. Effects of Different Treatments on Soil Microbial Biomass and Physicochemical Properties

**Figure 4:**
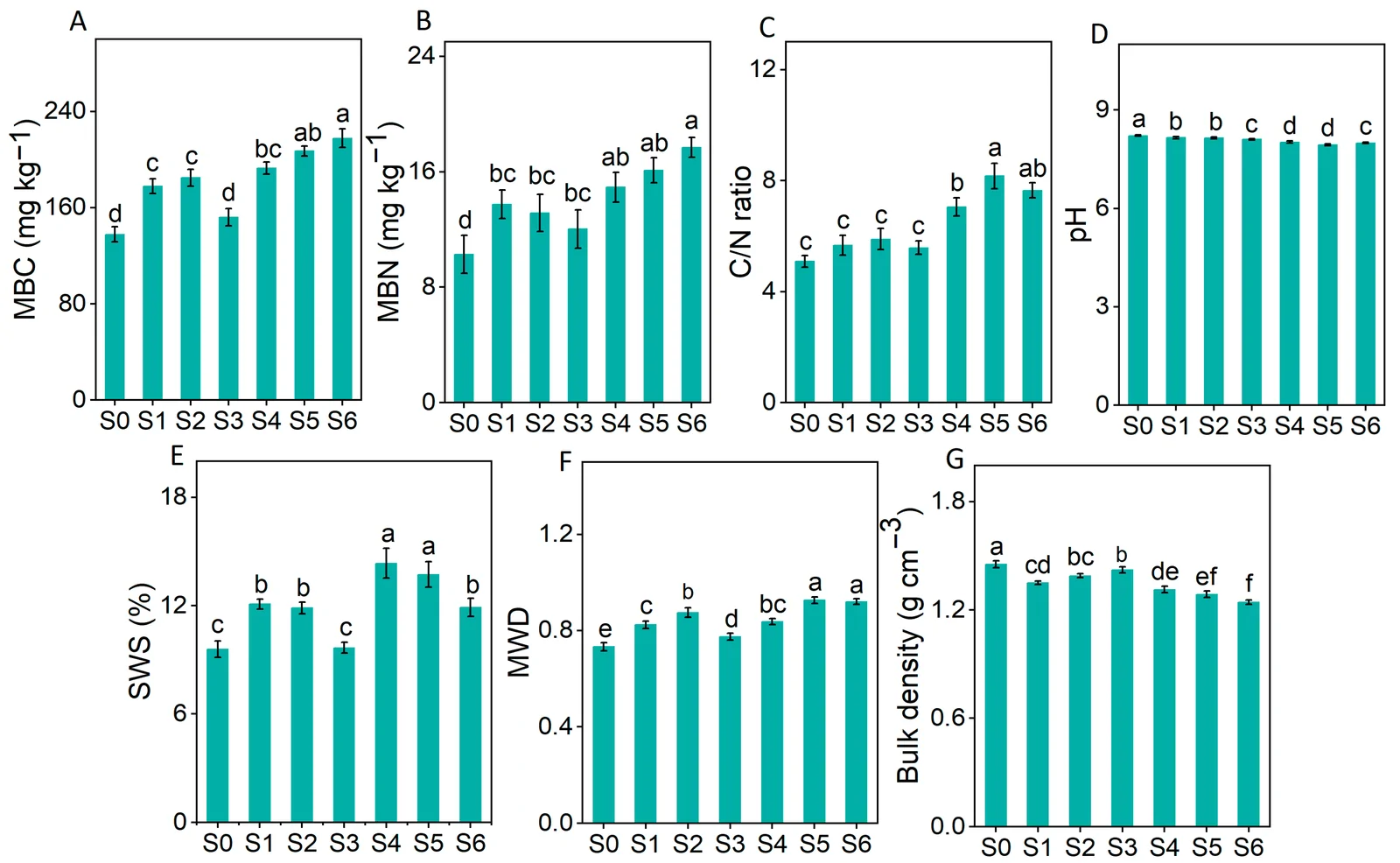
Soil microbial biomass carbon (MBC) (**A**), microbial biomass nitrogen (MBN) (**B**), microbial C/N ratio (**C**), pH (**D**), soil water content (SWC) (**E**), mean weight diameter (MWD) (**F**), and bulk density (**G**) under the different treatments in the 2024 growing season. Different lowercase letters indicate significant differences among the seven treatments (*p* < 0.05, *n* = 3).

Vegetation configuration significantly regulated soil microbial biomass carbon (MBC) and microbial biomass nitrogen (MBN), with consistent divergent patterns detected between monoculture and mixed-cropping treatments (*p* < 0.05; Figure 4). The unseeded control (S0) yielded the lowest MBC concentration (137.80 mg kg^−1^), while the mixed-cropping S6 treatment produced the maximum MBC value (217.81 mg kg^−1^). Collectively, monoculture assemblages exhibited lower MBC levels (152.10–177.90 mg kg^−1^) relative to mixed-cropping communities (193.85–217.81 mg kg^−1^). MBN exhibited a consistent variation trend with MBC across all treatments. Mixed-cropping treatments recorded significantly higher MBN concentrations (14.93–17.68 mg kg^−1^) than monoculture treatments (12.03–13.75 mg kg^−1^), and the unseeded control maintained the minimum MBN content (10.28 mg kg^−1^).

No significant differences in soil microbial C/N ratio were observed among monoculture treatments and the unseeded control. The mixed-cropping S5 treatment had the highest microbial C/N ratio (8.17), which was statistically higher than that of all other treatments. Soil pH varied within a narrow range yet differed significantly among treatments (Figure 4D), being highest in the unseeded control and slightly lower in the mixed-cropping treatments. Soil water content (SWC) and mean weight diameter (MWD) exhibited identical variation patterns, both of which were generally higher in mixed-cropping treatments than in monocultures and the unseeded control. By comparison, soil bulk density showed an opposite trend. The unseeded control (S0) had the highest bulk density (1.45 g cm^−3^), followed by monoculture treatments (1.35–1.42 g cm^−3^), whereas mixed-cropping assemblages presented relatively lower bulk density values (1.24–1.31 g cm^−3^). In general, mixed cropping corresponded with increased microbial biomass accumulation, improved soil water retention and aggregate stability, and reduced soil compaction compared with the monoculture and unseeded control treatments. These observable variations indicate that vegetation composition exerts consistent regulatory effects on soil microbial characteristics and physical properties, reflecting that a reasonable species combination can optimize basic soil conditions in alpine vegetation restoration.

### 3.4. Effects of Different Treatments on Soil Characteristics and Plant (Aboveground and Belowground) Biomass

**Figure 5:**
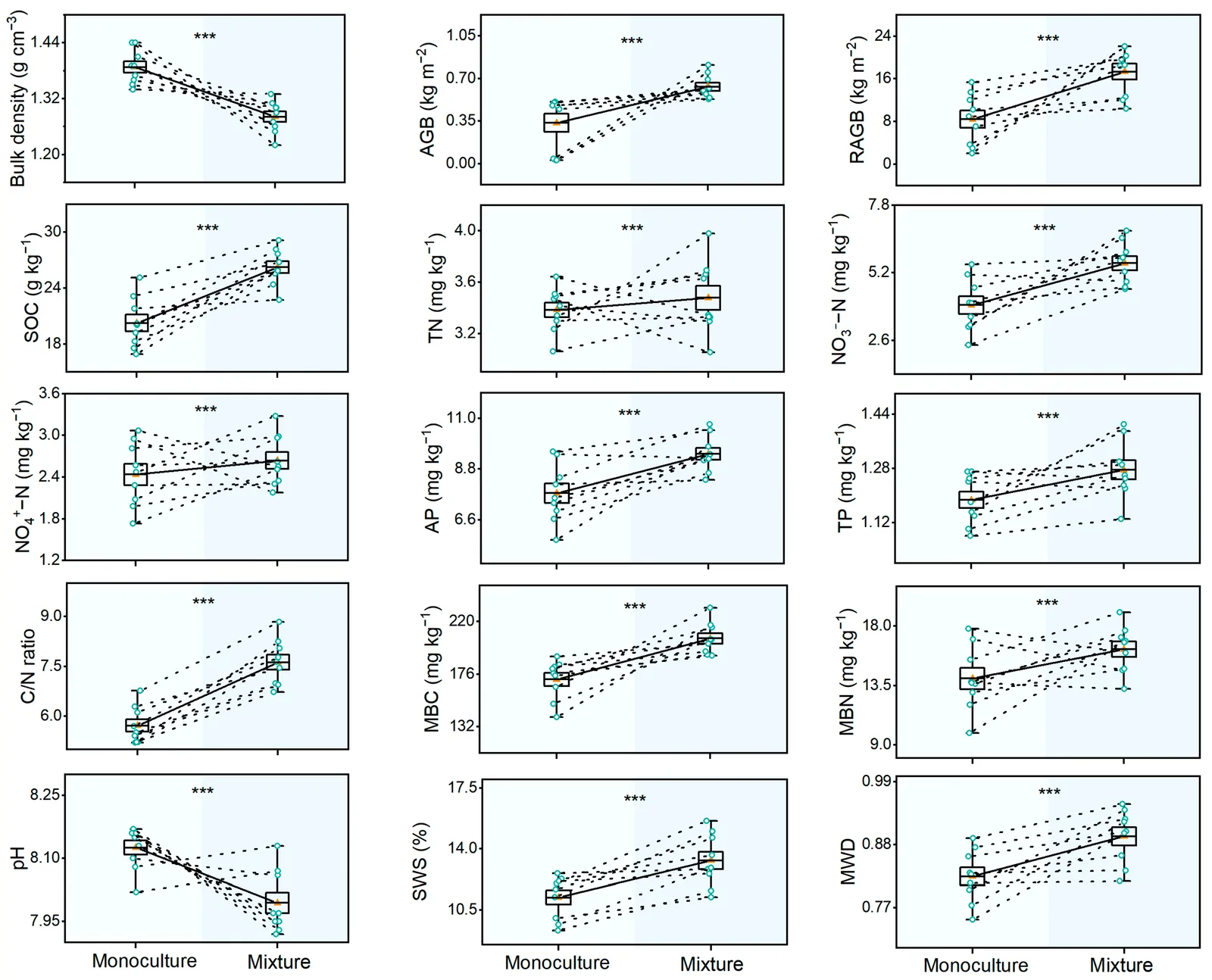
Comparison of plant and soil properties between the monoculture and mixed-cropping groups. Differences between the two groups were examined using independent−samples *t*-tests (*p* < 0.05). Boxes represent the interquartile range with the median line, whiskers represent the data range, dots represent individual plots, the orange triangle in the middle of each box represents the mean value of the data, and dashed lines connect paired observations across the two groups; the shaded background bands serve only as visual separation of variables. ***, *p* < 0.001.

Comparative analysis of plant biomass, soil physical properties, soil carbon and nutrient status, and soil microbial characteristics revealed significant differences between monoculture and mixed-cropping treatments. Most parameters reached their minimum values in the unseeded control (S0), which served as the degraded reference for these comparisons. Most plant and soil variables were greater under mixed-cropping regimes, while soil bulk density and pH were slightly lower (*p* < 0.05; Figure 5). Relative to monocultures, mixed-cropping treatments increased aboveground biomass, belowground biomass, soil organic carbon, total nitrogen, nitrate nitrogen, ammonium nitrogen, available phosphorus, total phosphorus, soil C/N ratio, microbial biomass carbon, microbial biomass nitrogen, soil water content, and mean weight diameter by 25%, 16%, 30%, 3%, 41%, 8%, 19%, 9%, 38%, 26%, 10%, 19%, and 12%, respectively. Soil bulk density and pH declined moderately by 8% and 1%, respectively. It should be noted that this pooled contrast combines treatments that differ in life form, method of introduction, and time of establishment; it is therefore used only as a descriptive summary, and the treatment-level analysis remains the primary basis for statistical inference.

### 3.5. Correlations Between the Plant Facilitation Index and the Soil Fertility Index

**Figure 6:**
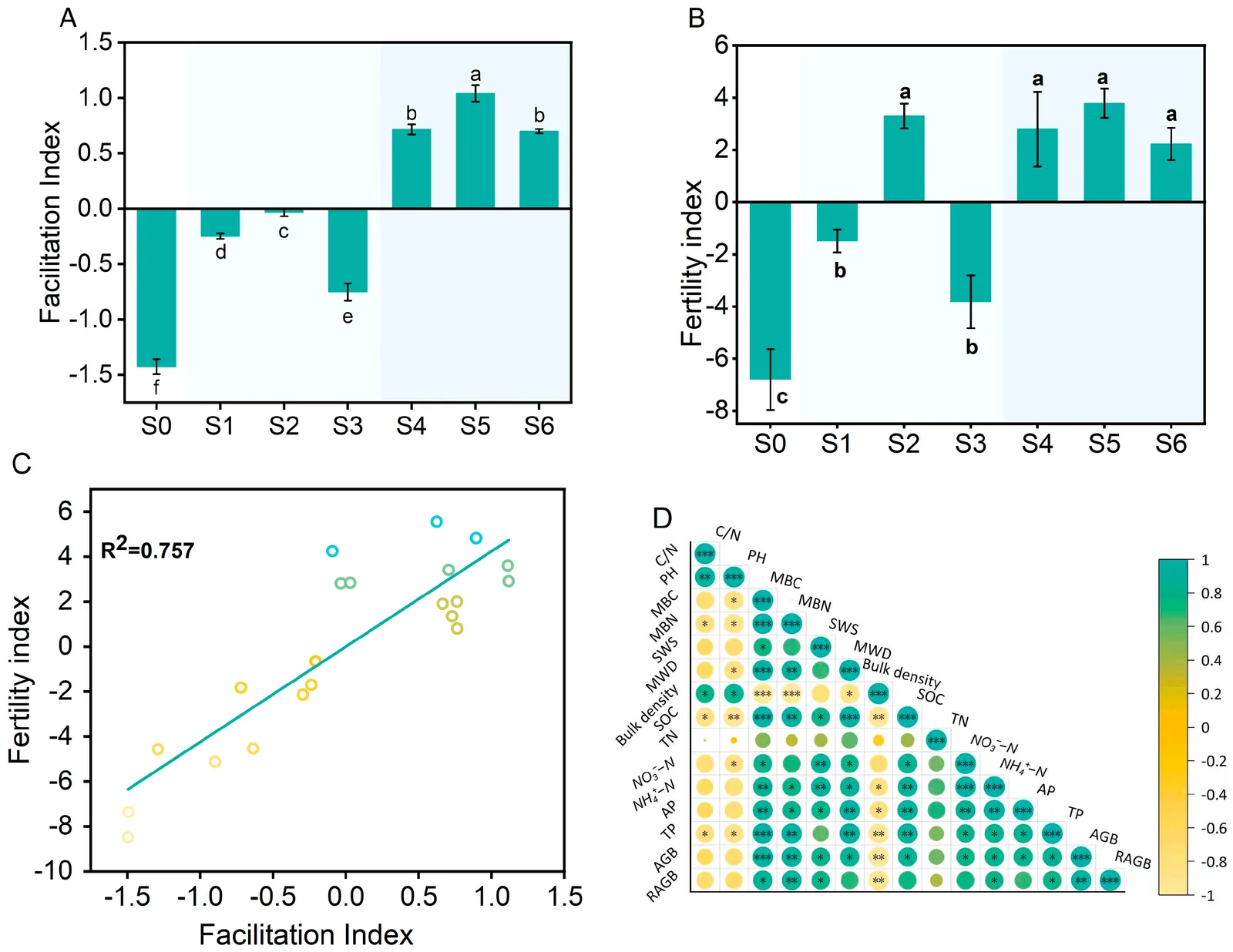
Facilitation index under the different sowing treatments in the 2024 growing season (**A**); comprehensive soil fertility index under the different sowing treatments (**B**); linear relationship between the facilitation index and the fertility index (**C**); and Pearson correlation analysis of plant and soil variables under the different treatments (**D**). In (**D**), circle colors represent the Pearson correlation coefficients according to the color scale. *, *p* ≤ 0.05; **, *p* ≤ 0.01; ***, *p* ≤ 0.001. Different lowercase letters indicate significant differences among treatments.

The plant facilitation index differed significantly among vegetation treatments (Figure 6A). The unseeded control and the monoculture assemblages produced negative facilitation values (−1.43 to −0.05), whereas all mixed-cropping treatments yielded positive values (0.70 to 1.01). The comprehensive soil fertility index also exhibited pronounced treatment-dependent variation. The unseeded control (S0) recorded the lowest fertility index (approximately −6.7), which was significantly lower than that of all vegetated treatments. The monoculture treatments S1 and S3 also showed negative fertility values of −1.5 and −3.8, indicating poor soil fertility, though their values were less negative than S0. In comparison, treatments S2, S4, S5, and S6 exhibited positive fertility values of 3.3, 2.8, 3.8, and 2.2. These four treatments showed statistically comparable fertility levels (*p* > 0.05).

Regression analysis identified a significant positive relationship between facilitation index and soil fertility index (R^2^ = 0.757), indicating that greater interspecific facilitation was closely associated with higher soil fertility (Figure 6C). Pearson correlation analysis further demonstrated consistent covariation among soil physicochemical, microbial, and vegetative variables. Both aboveground and root biomass were positively correlated with key soil functional indicators, including SOC, TN, MBC, MBN, soil water content, MWD, nitrate nitrogen, ammonium nitrogen, available phosphorus, and total phosphorus. In contrast, soil bulk density, microbial C/N ratio, and soil pH were negatively correlated with most fertility-related parameters.

In summary, the unseeded control and most monoculture treatments corresponded with lower or negative soil fertility status, whereas the mixed-cropping assemblages generally sustained positive plant facilitation and relatively improved soil fertility; the positive fertility index of the monoculture S2 indicates, however, that this pattern was treatment-specific rather than a simple monoculture-versus-mixture contrast. The facilitation index was significantly correlated with plant productivity and with several soil physicochemical and microbial properties, indicating that the species interactions estimated by the index were associated with soil functional characteristics; because facilitation was assessed through an index and the relationships are correlational, these results do not by themselves demonstrate a causal regulatory mechanism. Under the conditions of this single-season experiment, some species combinations were associated with more favorable plant–soil responses than others; whether these combinations also enhance the long-term stability of restored vegetation communities cannot be inferred from the present data and requires long-term monitoring.

## 4. Discussion

### 4.1. Effects of Different Treatments on Plant Aboveground and Belowground Biomass

The restoration of degraded alpine grasslands on the Qinghai–Xizang Plateau requires optimized vegetation allocation and sustained improvement in soil functional performance. Plant interspecific interactions are fundamental biotic drivers of community assembly and ecosystem functioning; however, the facilitative mechanisms governing mixed-cropping vegetation establishment remain insufficiently clarified in alpine restoration research. This study showed that the mixed-cropping assemblages S4 and S6 accumulated significantly greater aboveground and belowground biomass than the monoculture treatments. Such productivity advantages may reflect several non-exclusive mechanisms—including functional complementarity, interspecific facilitation, selection effects, and differences in sowing density and establishment rates—which could not be separated in the present study. Notably, soil microbial communities are increasingly recognized as key regulators of soil functions and ecosystem multifunctionality in alpine grasslands [33].

Functional differentiation between grass and legume species may underpin the biomass overyielding observed in the mixed-cropping systems. As a legume, *Medicago sativa* is capable of symbiotic nitrogen fixation, which may have increased soil available nitrogen and partially relieved the nitrogen limitation of the companion grass *Elymus breviaristatus*; however, nodulation, nitrogen-fixation rates, and species-specific plant nitrogen uptake were not measured in this study, so this mechanism is inferred from the literature rather than demonstrated. In turn, *Medicago sativa* develops a deep taproot, whereas *Elymus breviaristatus* forms a dense, more surficial fibrous root system; this contrast in rooting patterns may have reduced belowground competition, although root-depth distributions were not measured. Beyond nitrogen supplementation, root exudates released by *Medicago sativa* modulate rhizosphere pH and mobilize insoluble phosphorus fractions, further improving soil phosphorus availability for neighboring plants. Consistent with previous evidence, legume-derived nitrogen inputs can enhance the leaf nitrogen status and photosynthetic potential of grasses, supporting continuous vegetative growth. In this study, all treatments containing *Medicago sativa* (S2, S5, S6) exhibited elevated soil nitrogen and phosphorus concentrations. These results are consistent with the interpretation that legume incorporation improves soil nutrient availability and thereby supports aboveground biomass accumulation in alpine plant communities.

*Tortula subulata* incorporation may further modulate microhabitat conditions and enhance community performance through ecological engineering effects. Surface *Tortula subulata* cover may stabilize near-ground microclimates and improve soil water retention, which is consistent with the higher soil water content observed in the moss-containing treatments; evaporation itself was not measured in this study. These microenvironmental modifications may also enhance soil aggregate stability [34,35] and support a greater soil microbial biomass; respiration or enzyme assays would be required to assess microbial metabolic activity directly. Correspondingly, all *Tortula subulata*-containing mixed-cropping treatments exhibited improved root development, higher soil water content, and lower soil bulk density compared with their corresponding monocultures. These observations indicate that *Tortula subulata*-mediated amelioration of soil physical and hydrological properties creates favorable belowground environments for the coordinated growth of *Elymus breviaristatus* and *Medicago sativa*. Furthermore, partial overlap in water resource demand between grass and legume species may induce moderate interspecific competition. Such mild competitive stress promotes vertical root proliferation and increases belowground carbon allocation, optimizing root spatial distribution and resource acquisition capacity. In other words, the association improves nutrient availability for the component species through a balance between moderate competition and the complementary use of different soil layers and nutrient forms; this nutrient partitioning among species allows the partners to coexist, because competition remains too weak to cause the exclusion of any one species. This pattern is consistent with niche complementarity and with the “conscious complementarity” described for grass–legume associations, in which the legume’s fixation of atmospheric nitrogen ultimately serves its own survival under rapid nutrient depletion by the dominant grass, while simultaneously acting as a “nitrogen pool” for the companion grass [20,21]. It should be emphasized, however, that nutrient partitioning and complementarity effects were inferred from treatment-level patterns rather than measured directly, and contributions from species identity effects, selection effects, and microenvironmental changes cannot be excluded.

Overall, the biomass superiority of mixed-cropping communities derives from the synergistic combination of interspecific facilitation and moderate resource competition. Species functional complementarity improves soil nutrient supply and hydrological conditions, while moderate competitive pressure optimizes belowground resource allocation, jointly promoting coordinated above- and belowground plant growth. The consistent covariation of plant productivity, soil physicochemical attributes, and microbial parameters suggests that vegetation composition and species interactions are jointly associated with soil functional characteristics in alpine ecosystems; similar positive effects of legume-based diversification on soil nitrogen and phosphorus availability and microbial properties have been reported in alfalfa-based systems and in monoculture experiments [36,37]. For the restoration of degraded alpine grasslands, rationally configured mixed-cropping vegetation can maximize facilitative interactions, reduce excessive competitive exclusion, and improve community productivity and soil quality. This study provides a candidate vegetation allocation strategy for sustaining the stability of restored alpine ecosystems and maintaining soil functional sustainability under high-altitude environmental constraints.

### 4.2. Effects of Different Treatments on Soil Fertility

Mixed cropping yielded an average SOC concentration of 26.24 g kg^−1^, 30% higher than that measured under monoculture. This observation aligns with existing evidence that diversified plant communities promote soil carbon sequestration [38,39,40,41,42]. Two mechanisms account for this improvement. First, mixed vegetation supplies abundant and continuous organic substrates to soil carbon pools via diversified root exudates and greater litter inputs. Second, interspecific functional complementarity sustains carbon inputs throughout the growing season and buffers seasonal fluctuations in carbon supply commonly found in monoculture systems. Although symbiotic nitrogen fixation by *Medicago sativa* was not measured directly and total nitrogen (TN) did not increase significantly, the mixed-cropping treatments showed markedly elevated soil nitrate nitrogen concentrations (4.82–6.33 mg kg^−1^). This pattern is consistent with faster soil nitrogen transformation under mixed cropping; however, because nitrifier communities were not measured and nitrate reflects the net balance of several processes, this interpretation remains tentative, and elevated nitrate with unchanged TN may also indicate a potential risk of nitrogen loss.

### 4.3. Effects of Different Treatments on Soil Microbial, Chemical, and Physical Properties

Soil physical structural improvement is a fundamental mechanism by which mixed-cropping regimes modulate edaphic fertility and ecosystem functionality in alpine grasslands. Soil structural properties and soil microbial biomass interactively influence grassland ecosystem functioning, and such coupled processes are particularly critical for the Qinghai–Xizang Plateau, where fragile and climate-sensitive soils act as primary constraints on the recovery of degraded grassland landscapes. The present study indicated that mixed-cropping assemblages significantly ameliorated soil physical quality. The average soil bulk density under mixed cropping was 1.28 g cm^−3^, which was 8% lower than that under monoculture (1.39 g cm^−3^) and substantially lower than that of the unseeded control (1.45 g cm^−3^). Correspondingly, soil mean weight diameter (MWD) increased by 12% relative to monoculture treatments. These structural variations may be associated with belowground functional complementarity and root niche partitioning between coexisting species, although these processes were not directly measured. The deep taproot of *Medicago sativa* can penetrate below the 0–20 cm sampling layer, whereas the fine fibrous roots of *Elymus breviaristatus* are concentrated in the topsoil; root sampling in this study was restricted to the 0–20 cm layer. Such stratified rooting may enhance soil pore connectivity, and root-derived polysaccharides may promote the formation of stable microaggregates [34,35]. This series of biotic modifications mitigates soil compaction and alleviates soil hardening caused by frequent freeze–thaw cycles, thereby reinforcing the structural stability of alpine soils.

Optimized soil physical configurations reshape subsurface aeration and hydrological regimes, constructing a habitable microenvironment for soil microbial proliferation and metabolism. Relative to monoculture planting, mixed-cropping treatments increased soil microbial biomass carbon (MBC) and nitrogen (MBN) by 26% and 10%, with peak values of 217.81 mg kg^−1^ and 17.68 mg kg^−1^, respectively. The *Elymus breviaristatus* and *Medicago sativa* mixed treatment (S6) exhibited the highest MBC concentration, which is consistent with a positive feedback cascade in which improved soil structure facilitates belowground carbon input and thereby supports microbial biomass accumulation. Moreover, the microbial C/N ratio of the *Medicago sativa*–*Tortula subulata* mixed treatment (S5; 8.17) fell within the optimal stoichiometric range (8–12) reported for soil microbial communities. The balanced microbial C/N stoichiometry is conducive to optimizing microbial community structure and sustaining high-efficiency soil nutrient cycling in alpine soils.

Soil pH exhibited significant divergent patterns between monoculture and mixed-cropping systems, owing to interspecific differences in rhizosphere biochemical regulation. *Medicago sativa* secretes organic acids (e.g., oxalic acid) to mobilize insoluble soil phosphorus, which moderately reduces rhizosphere pH. In contrast, monocultured grass species release alkaline metabolites that buffer rhizosphere fluctuations and maintain relatively stable soil pH. Such species-specific rhizosphere modulation reorganizes soil microbial community assembly and accelerates the transformation and turnover of soil nutrients. In conclusion, mixed-cropping vegetation triggers synergistic optimization of soil physical structure, rhizosphere biochemical properties, and microbial functional traits. The coordinated amelioration of multi-dimensional edaphic attributes alleviates the intrinsic soil limitations of alpine ecosystems, providing a stable and sustainable soil foundation for the ecological restoration of degraded Qinghai–Xizang Plateau grasslands.

### 4.4. Integrated Comparison of Soil Characteristics and Plant Biomass Between Monoculture and Mixed-Cropping Systems

When the treatments were pooled into monoculture and mixed-cropping groups, most plant and soil variables differed significantly between the two groups (Figure 5): aboveground biomass, belowground biomass, SOC, TN, nitrate nitrogen, ammonium nitrogen, available phosphorus, total phosphorus, soil C/N ratio, MBC, MBN, soil water content, and MWD were higher under mixed cropping by 3–41%, whereas bulk density and pH were slightly lower (by 8% and 1%, respectively). These pooled differences are consistent with the treatment-level analyses and indicate an overall positive association between mixed cropping and the measured plant–soil variables. However, because the pooled contrast combines treatments that differ in life form, method of introduction, and time of establishment, it should be interpreted as descriptive, and mechanistic inference should rely on the treatment-level results. Finally, because this experiment covered a single growing season, potential year effects, which are common in mixed-cropping systems, could not be assessed; multi-year, multi-site experiments are therefore identified as an essential direction for future research.

### 4.5. Relationship Between Interspecific Facilitation and Soil Fertility

The strong positive correlation between the facilitation index and soil fertility index (R^2^ = 0.757) suggests that interspecific facilitation is closely associated with the beneficial effects of mixed cropping, although causality cannot be established from this correlation alone. The advantages of species mixing operate across three scales. At the plant scale, niche partitioning between deep-rooted and shallow-rooted species (*Elymus breviaristatus* and *Medicago sativa*, for example) reduces direct belowground resource competition. At the microbial scale, synergistic interactions between legume-associated rhizobia and plant growth-promoting rhizobacteria of grasses may facilitate rhizospheric nutrient mobilization [43,44]. At the soil scale, accelerated root turnover fosters soil aggregate formation, which physically protects soil carbon and nitrogen from loss. Among all treatments, the mixture of *Elymus breviaristatus* and *Medicago sativa* exhibited the strongest positive effects, with the highest aboveground biomass, SOC, and microbial biomass carbon (MBC), consistent with intense interspecific facilitation. By comparison, the combination of *Elymus breviaristatus* and *Tortula subulata* produced low root biomass and only slight SOC accumulation, presumably due to resource competition between shallow moss rhizoids and grass roots. These contrasting outcomes demonstrate that the performance of mixed-cropping systems depends largely on the degree of functional trait complementarity among co-occurring species [45,46]. Therefore, selecting species with evident niche differentiation (e.g., deep-versus shallow-rooted species, nitrogen-fixing versus non-nitrogen-fixing species) is critical for improving soil fertility and sustaining ecosystem multifunctionality.

The prominent difference in soil fertility index between the degraded control (S0: −6.7) and optimal mixed-cropping treatment (S5: 3.8) indicates that, under the present experimental conditions, mixed cropping contributed to the recovery of soil fertility within a single growing season; whether this effect represents a durable reversal of degradation requires longer-term observation. In addition to boosting vegetation productivity, mixed cropping comprehensively improves soil fertility by optimizing soil physical structure and supporting soil microbial biomass. These single-year results suggest that the grass–legume–moss association could serve as a potential model of favorable species interaction and that trait-based species combination offers a promising ecological approach for restoring degraded grasslands across the Qinghai–Xizang Plateau; however, strong conclusions should not be drawn from a single year of data, and multi-year validation is required.

## 5. Conclusions

In this single-season field experiment in an alpine artificial grassland on the Qinghai–Xizang Plateau, mixed cropping increased above- and belowground biomass, soil organic carbon, nitrate nitrogen, and soil microbial biomass, and reduced soil bulk density relative to most monocultures and the unseeded control, whereas the effects on total nitrogen, total phosphorus, and available phosphorus were small or significant only for individual treatments [19,38,39]. The facilitation index was positively correlated with the comprehensive soil fertility index (R^2^ = 0.757), indicating that the plant-plant interactions estimated by the index were associated with the improvement of soil fertility [18,20]; however, facilitation was not measured directly, and functional complementarity, selection effects, and differences in sowing density remain plausible alternative explanations.

Because the experiment was conducted at a single site over one growing season with three replicates per treatment, these findings provide preliminary, short-term evidence that mixed cropping is a potentially useful approach for the restoration of degraded alpine meadows [7,8,12] rather than a validated in situ restoration strategy. Multi-site, multi-year experiments with direct measurements of nitrogen fixation, functional traits, root distributions, and microbial activity are needed to confirm the underlying mechanisms and to evaluate the broader applicability of mixed cropping for alpine grassland conservation.

## Data Availability

The original contributions presented in this study are included in the article. Further inquiries can be directed to the corresponding author.
